# Relative Deficiency of Anti-Inflammatory *N*-Acylethanolamines Compared to Prostaglandins in Oral Lichen Planus

**DOI:** 10.3390/biomedicines8110481

**Published:** 2020-11-06

**Authors:** Linda Rankin, Sandra Gouveia-Figueira, Karin P. Danielsson, Christopher J. Fowler

**Affiliations:** 1 Department of Integrative Medical Biology, Faculty of Medicine, Umeå University, SE-901 87 Umeå, Sweden; linda.rankin@umu.se; 2 Clinical Chemistry, Region Västerbotten, SE-901 85 Umeå, Sweden; sandra.gouveia@regionvasterbotten.se; 3 Department of Odontology, Faculty of Medicine, Umeå University, SE-901 87 Umeå, Sweden; karin.p.danielsson@umu.se; 4 Department of Medical Biosciences, Faculty of Medicine, Umeå University, SE-901 87 Umeå, Sweden

**Keywords:** oral lichen planus, palmitoylethanolamide, *N*-acylethanolamine, prostaglandin, cyclooxygenase-2

## Abstract

Oral lichen planus (OLP) is a chronic inflammatory oromucosal disease. The *N*-acylethanolamines (NAEs), are a family of endogenous biologically active lipid mediators, with palmitoylethanolamide (PEA) being of particular interest here due to its anti-inflammatory and analgesic properties. In this study using oral mucosa biopsies from OLP patients and healthy controls, we investigated whether NAE synthesis was mobilized in response to the inflammation associated with OLP. *PTGS2* levels, coding for cyclooxygenase-2 (COX-2), were increased approximately 4-fold in OLP compared to controls and a significant increase in the ratio of *PTGS2* to *NAPEPLD*, the latter coding for a key enzyme in NAE synthesis, was seen. This was matched by an increased ratio of COX-2-derived prostaglandins to PEA in a second patient cohort. We conclude that there is an imbalance between prostaglandins and PEA in OLP, opening up the possibility that PEA might be a useful treatment for this disorder.

## 1. Introduction

Oral lichen planus (OLP) is a common chronic inflammatory disease affecting the oral mucosa, where it forms lesions that are troublesome for those affected. OLP is a subtype of lichen planus, a family of inflammatory diseases affecting the skin in various areas, commonly genitalia and mucosa [1]. The prevalence of OLP has been estimated to 1–2% worldwide, and women are predominantly affected over men [2]. The etiology of OLP, which has periods of improvement and relapses, is currently unknown, but involves, among other factors, activated cytotoxic CD8^+^ T cells, metalloproteinase activation, an influx of mast cells and macrophages accompanied by activation of NF-κß and increased levels of the cytokines interleukin-4 (IL-4), IL-6, IL-8, interferon-γ, and tumor necrosis factor-α (TNF-α) [1,2,3]. The expression of cyclooxygenase-2 (COX-2), responsible for production of prostaglandins (PGs) in inflammatory tissues, is upregulated in OLP [4,5,6,7] and has been suggested to be part of the proposed autoimmune character of the disease [6]. Although the first-in-line treatment option for OLP is topical (or sometimes systemic) steroids, their efficacy is variable and there is a clear need for novel treatment strategies.

*N*-acylethanolamines (NAEs) are a family of endogenous lipids that are primarily synthesized from membrane lipids via *N*-acylphosphatidylethanolamines (NAPEs). The final step of the synthesis, NAPE ⟶ NAE, is catalyzed by the enzyme NAPE phospholipase D (NAPE-PLD) [8]. In most tissues, the relative amounts of the different NAEs reflect the concentrations of the original membrane lipids, with palmitoylethanolamide (PEA), stearoylethanolamide (SEA) and oleoylethanolamide (OEA) being predominant [9]. These compounds all have biological activities: arachidonoylethanolamine (AEA, anandamide), a minor component of the NAEs, is an endogenous cannabinoid receptor ligand [10], whilst OEA acts as an endogenous satiety agent [11]. PEA has anti-inflammatory properties, thought to be mediated primarily via peroxisome proliferator-activated receptor α (PPAR-α), resulting in the inhibition of NF-κß and thereby the downstream release of pro-inflammatory cytokines [12,13]. PEA reduces COX-2 expression in a mouse model of acute inflammation [14], reduces PG levels in activated RAW 264.7 macrophages in vitro without per se affecting the expression of either mRNA for *PTGS2* (which codes for COX-2) or the protein itself [15], and downmodulates mast cells in vivo [16]. In humans, PEA is well tolerated and shows both analgesic efficacy when given systemically (for review, see [17]) and efficacy for the topical treatment of eczema, where it considerably reduces the need for glucocorticoid treatment [18]. NAEs are hydrolyzed to their corresponding long-chain fatty acids by the enzymes fatty acid amide hydrolase (FAAH) and *N*-acylethanolamine acid amidase (NAAA) [8], and inhibitors of these enzymes produce anti-inflammatory effects in experimental animals (see, e.g., [19,20]).

Given the anti-inflammatory properties of PEA, an attractive hypothesis is that it plays a role in either the pathogenesis of inflammatory disorders (where a relative deficiency fails to keep the inflammation in check) or as a response to the disorder to mitigate the level of inflammation. Most work in this respect has been undertaken in animal models, but a decreased level of PEA in synovial fluid from patients with rheumatoid arthritis and osteoarthritis has been reported [21], whilst changes in the relative expressions of FAAH and NAAA have been reported in patients with ulcerative colitis [22]. Very little is known about PEA and other NAE levels in human oral disorders, the only study to our knowledge being that of Barry et al. [23] who reported that plasma levels of PEA, but not of OEA or AEA, were increased in patients with burning mouth syndrome, but these authors did not investigate local changes in the mouth. Consequently, in the present study, we have investigated the balance between PEA and COX-2-derived prostaglandins in biopsy samples from patients with OLP.

## 2. Materials and Methods

### 2.1. Tissue Biopsy Samples

Punch biopsies (4 mm) from buccal mucosa were collected after obtaining informed consent from volunteers without OLP (“controls”) and from patients clinically and histologically diagnosed with OLP. All OLP biopsies were taken from reticular areas and were collected between June 2012 and December 2018. Neither controls nor patients were medicated with immunosuppressors or non-steroidal anti-inflammatory drugs at the time of biopsy. All patients with OLP had more or less symptomatic lesions at the time. OLP was diagnosed according to the modified World Health Organization (WHO) diagnostic criteria (which includes histopathological verification), as described in [24]. None of the patients had a diagnosis of paradontitis. Unless otherwise stated, the samples were embedded in Tissue-Tek, snap frozen in liquid nitrogen and thereafter stored at −80 °C. This study was approved by the ethical review board at Umeå University (Dnr 09-083M). All experiments for this study were performed in accordance with the declaration of Helsinki and European Union regulations. Patient data were anonymized and informed consent was obtained from the patients prior to participation.

Two cohorts were used. For the “qPCR cohort”, biopsies were obtained from 30 volunteers: 15 OLP patients (10 females, 5 males; age range 44–81 years, median 64 years) and 15 controls (12 females, 3 males; age range 39–73 years, median 61 years). Details of the cases in the qPCR cohort are provided in Table 1. The two groups did not differ significantly with respect to age (*p* = 0.30, Mann–Whitney U test) or gender distribution (*p* = 0.68, Fisher’s exact test). None of the patients in the cohort smoked, although one patient used moist powder tobacco (Swedish snuff), and none of the patients had hepatitis C. For the “PG/NAE cohort”, biopsies were obtained from 8 patients with OLP (4 females, 4 males; age range 43–79 years, median 61 years) and 8 volunteers without OLP (6 females, 2 males; age range 43–67 years, median 50 years; *p* value for control vs. OLP age 0.49, Mann–Whitney U test). Four of the OLP biopsies and 7 of the control samples in the “PG/NAE cohort” have previously been described with respect to an Illumina Gene expression study [24] and the linoleic acid oxylipin derivatives for this cohort have been reported in [25]. With respect to sample sizes, a formal a priori power calculation is difficult given that there is no available information upon NAEs in OLP, but we have instead looked for a consistent picture of results in the two cohorts used.

### 2.2. RNA Extraction

Biopsies were stored in −80 °C prior to RNA extraction. For samples embedded in Tissue-Tek, two scalpels were used to cut the tissue out as it was thawing. The remaining samples had been frozen directly in the vial. Homogenization was undertaken using the Precellys 2 mL soft tissue homogenizing ceramic beads kit (CK14, Bertin Instruments, Montigny-le-Bretonneux, France) and Precellys 24 tissue homogenizer. The tissue was cut into quarters using a scalpel and then placed in the Precellys tubes, adding 600 µL kit buffer RLT Plus followed by placing tubes on ice. Aliquots (600 µL) of homogenized sample were used with the Qiagen Allprep DNA/RNA/miRNA kit (Qiagen, Hilden, Germany) and extraction was performed according to supplier instructions. The extracted RNA was eluted once and quality control was performed using NanoDrop Lite (Thermo Fisher Scientific, Waltham, MA, USA) whereupon extracted samples were placed in −20 °C. The GoScript reverse transcription mix, Oligo(dT) (Promega, Madison, WI, USA), was used for the conversion of RNA from the biopsy samples, diluted to contain 15 ng/µL total RNA, into cDNA and the reverse transcription was performed in the LifeECO thermal cycler (BIOER, Hangzhou, China). The following conditions were used: 10 min at 25 °C, 120 min at 37 °C, followed by termination at 85 °C for 5 min and then cooling of the samples.

### 2.3. Treatment of CAL27 and SCC-25 Human Squamous Carcinoma Cells with TNF-α, IL-8 and PEA

CAL27 and SCC-25 cells (ECACC, Porton Down, UK, passage numbers 61–62 and 30–31, respectively) were maintained at 37 °C, with 5% CO_2_, in T75 cm^2^ flasks with in ATCC DMEM 30-2002 and 10% fetal bovine serum. The cells (250,000 cells per well, in 24-well plates) were treated with vehicle (V; DMSO, final concentration 0.3%), recombinant human TNF-α (R&D systems, Abingdon, UK; 10 ng/mL final concentration), IL-8 (R&D systems; 10 or 100 ng/mL final concentrations) and/or PEA (Cayman Chemical Co., Ann Arbor, MI, USA, 0, 3 or 10 µM final concentrations) for 24 h in normal culture media with reduced fetal bovine serum concentration to 1%, prior to experiments. Following the incubation period, cells were washed with cold phosphate-buffered saline prior to addition of cold lysis/binding buffer (100 µM tris(hydroxymethyl)aminomethane (Tris), 500 µM LiCl, 1% lithium dodecyl sulphate, 5 mM dithiothreitol, pH 7.5) and frozen at −80 °C. mRNA was extracted using DYNABEADS mRNA direct kits (Thermo Fisher Scientific) according to the manufacturer’s manual and reverse transcription was undertaken using high-capacity cDNA reverse transcription kits (Applied Biosystems, Thermo Fisher Scientific, Waltham, MA, USA). 

### 2.4. Real-Time qPCR (RT-qPCR)

For the biopsy samples, cDNA was undiluted except for the reference gene (*RPL19*) which was diluted 1:10. qPCR reactions were run using a SYBR green mix (KAPA SYBR FAST qPCR kit Master Mix, KAPA Biosystems, Wilmington, MA, USA) and an Eco™ real-time PCR system (Illumina Inc., San Diego, CA, USA) using the following protocol: initial holding time for 2 min at 95 °C, followed by 45 cycles of (5 s at 95 °C and 30 s at 60 °C) and a melt curve cycle of 15 s at 95 °C, 15 s at 55 °C and 95 °C for the final 15 s. Primers including their sequences and efficiencies, calculated using 6 sample serial dilutions over a 50-fold dilution range, are presented in Table 2. Efficiencies were not used to correct Ct values since they were all close to 100%. Results are presented as ΔCt with respect to the reference gene as this allows comparison of mRNA levels for the different genes.

### 2.5. Analysis of Oxylipins and N-Acylethanolamine (NAE) Derivatives

Levels of oxylipins and NAEs were quantitated using an ultra-performance liquid chromatography (UPLC) coupled to tandem mass spectrometry (MS/MS) method described in detail in [26]. Briefly, samples were spiked with oxylipin and NAE internal standard solutions (AEA-d_8_, PEA-d_4_, SEA-d_3_, OEA-d_4_, PGD_2_-d_4_, PGE_2_-d_4_, TXB_2_-d_4_, 20-HETEd_6_, 5(*S*)-HETE-d_8_, and 2-arachidonoylglycerol (2-AG)-d_8_, Cayman Chemical Co., Ann Arbor, MI, USA, for the data reported here), diluted in 1 mL 5% methanol in MilliQ distilled water and grounded using a bead mill (Retsch MM400; 30 oscillations/s for 2 min). After centrifugation, the supernatant was applied into solid-phase extraction Waters Oasis HLB cartridges (60 mg sorbent, 30 µm particle size) followed by elution with 2 mL methanol and 2 mL ethyl acetate into polypropylene tubes containing 6 µL glycerol solution (30% in methanol). The eluates were evaporated under vacuum and reconstituted in methanol containing 12-[[(cyclohexylamino)carbonyl]amino]-dodecanoic acid (Larodan, Malmö, Sweden) as a recovery standard.

UPLC-MS/MS analysis was performed immediately using a Waters BEH C18 column (2.1 × 150 mm, 2.5 µM particle size) and the mass analysis was performed on an Agilent 6490 Triple Quadrupole system equipped with the iFunnel Technology source (Agilent Technologies, Santa Clara, CA, USA) in the positive multiple reaction monitoring mode (for the NAEs, 2-AG and for *N*-arachidonoylglycine (NA-Gly)) and in the negative multiple reaction monitoring mode for the oxylipins. The internal standard recoveries were 106 ± 22% (mean ± SD), 120 ± 28%, 104 ± 25%, 107 ± 25%, 84 ± 9%, 71 ± 3%, 54 ± 14%, 88 ± 16%, 62 ± 10% and 107 ± 5% for AEA-d_8_, PEA-d_4_, SEA-d_3_, OEA-d_4_, PGD_2_-d_4_, PGE_2_-d_4_, TXB_2_-d_4_, 20-HETEd_6_, 5(*S*)-HETE-d_8_, and 2-AG-d_8_, respectively. In addition to the lipids reported here, seven linoleic acid derivatives were also detected for samples run in the positive multiple reaction monitoring mode. The data for these linoleic acid derivatives have been reported elsewhere [25].

### 2.6. Statistics

MANOVA was conducted using the stat package built into the R statistical software package (v. 3.5.1) [27]. Two-tailed *t*-tests not assuming equal variances were undertaken using the GraphPad Prism statistical software for the Macintosh (v. 8, GraphPad Software Inc., San Diego, CA, USA). A 5% false discovery rate [28] was used when multiple testing was employed. 

## 3. Results

### 3.1. mRNA Levels of NAPE, PTGS2, FAAH and NAAA in Control and OLP Biopsy Samples

**Figure 1:**
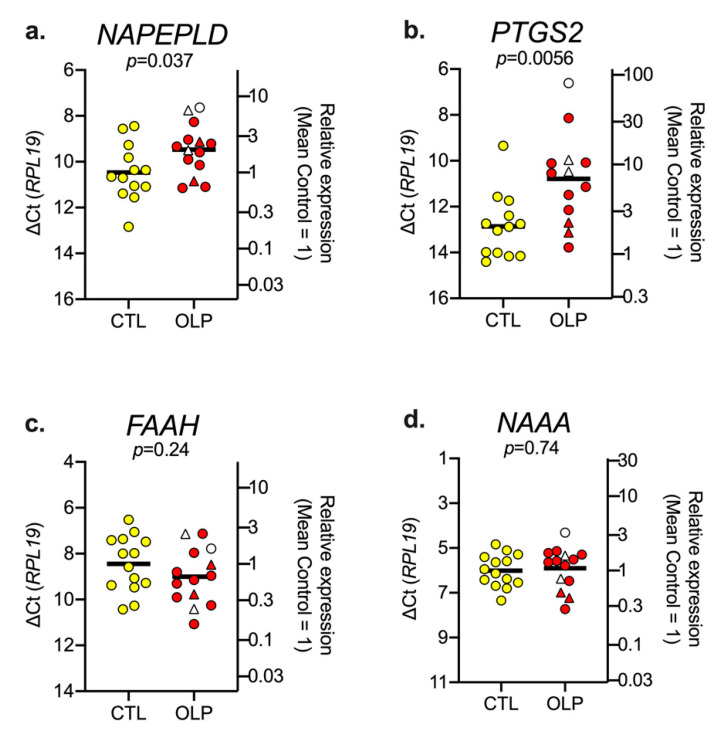
mRNA levels of (**a**) *NAPE*, (**b**) *PTGS2*, (**c**) *FAAH*, and (**d**) *NAAA* in biopsy samples from patients with OLP and healthy controls. The data are shown as scatter plots for the healthy controls (yellow circles, CTL) and patients with OLP; in both cases, *N* = 13–14. For OLP, the triangles are cases without accompanying genital/skin pathology, and the circles are those with accompanying genital/skin pathology. The unfilled symbols are cases without atrophic pathology; the red symbols are cases with atrophic pathology. The y axes have been chosen to give the same span of ∆Ct values for each mRNA. The bars show the mean values. For the ∆Ct values, a change of −1 indicates a doubling in the mRNA content and a change of +1 indicates a halving. The right y axes illustrate this with respect to the relative expression for the mean ∆Ct value for the controls. *p* values are for two-tailed *t*-tests not assuming equal variance. At a 5% false discovery rate, the critical value of *p* was 0.0125. In all cases, the residual plots were acceptable.

qPCR determinations were undertaken using biopsy samples from a cohort of 15 controls and 15 OLP patients (“qPCR cohort”) using *RPL19* as reference gene. Of these, there were insufficient tissue in l–2 cases, so the sample size was *N* = 13–14. The data are shown in Figure 1. 

OLP is a heterogeneous disorder, and cases can present with or without atrophic pathology. Further, cases can present with accompanying genital or skin lichen planus. In the PCR cohort samples that were analyzed, all but three cases showed atrophic pathology and all but four showed accompanying genital or skin lichen planus (see Table 1). The small number of cases with reticular pathology alone precludes an analysis of the different OLP subtypes, but we have indicated in Figure 1 whether the cases have atrophic pathology and accompanying genital or skin lichen planus. 

The levels of FAAH in the control biopsies were lower than the corresponding levels of NAAA (mean ∆Ct values of 8.45 vs. 6.01, corresponding to approximately a 5-fold difference in mRNA levels between the two. The mean ∆Ct value for *PTGS2* was significantly lower for the OLP cases (10.79, 95% CI 9.60–11.98, *N* = 13) than for the controls (12.86, 95% CL 12.01–13.71, *N* = 13, *p* = 0.0056, Welch’s *t*-test). The difference in the mean values (−2.07) corresponds to a quadrupling in the mRNA level. A significant increase was also seen for *NAPEPLD* (an approximate doubling in the mean mRNA content for the OLP cases compared to the controls), but the *p* value (0.037) was higher than the critical value of *p* of 0.0125 assuming a 5% false discovery rate. No significant changes in *FAAH* or *NAAA* mRNA levels were seen. 

**Figure 2:**
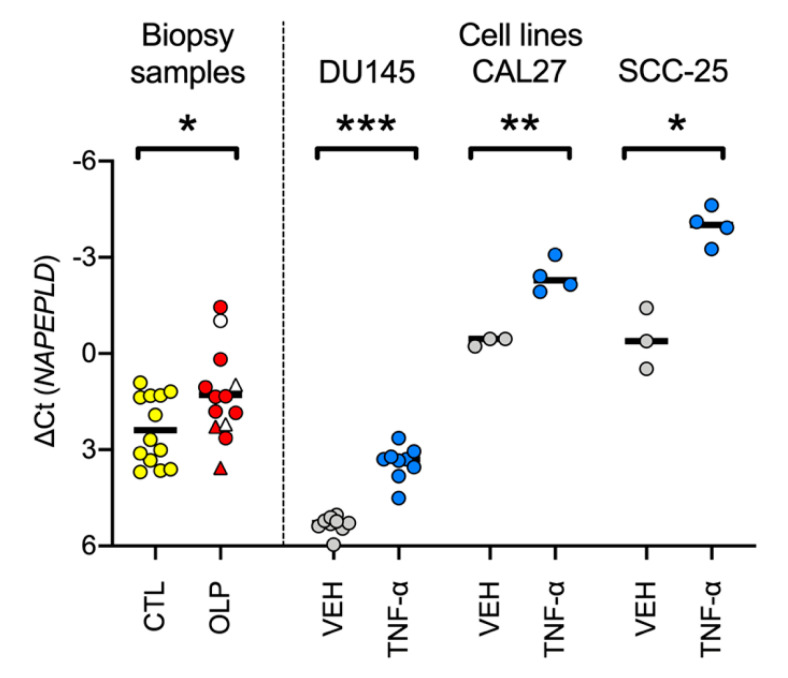
mRNA levels of *PTGS2* with *NAPEPLD* as reference gene in the biopsy samples and in three human cell lines treated with either vehicle or TNF-α. Control (CTL) biopsy samples are shown as yellow circles. For OLP, the triangles are cases without accompanying genital/skin pathology, and the circles are those with accompanying genital/skin pathology. The unfilled symbols are cases without atrophic pathology; the red symbols are cases with atrophic pathology. The data for the human DU145 prostate carcinoma cells are calculated from the raw data from Figure 1C of [29] using a two-hour exposure time to either vehicle (VEH, grey circles) or TNF-α (20 ng/mL, blue circles). The data for the two human squamous carcinoma cell lines (CAL27 and SCC-25) are calculated from the raw data shown in Supplemental Figures S1 and S2 and used a 24 h incubation time with either vehicle or TNF-α (10 ng/mL). The bars in the scatter plots represent the mean values. * *p* < 0.01, ** *p* < 0.01, and *** *p* < 0.001, two-tailed *t*-tests not assuming equal variance. At a 5% false discovery rate, the critical value of *p* was 0.05.

An alternative way of expressing the data, relevant to the “PG/NAE cohort” (see below), is to express the *PTGS2* data relative to *NAPEPLD* rather than to *RPL19* as reference gene, i.e., to express the data as a balance between a gene coding for the enzyme synthesizing inflammatory prostaglandins vs. a gene coding for an key synthetic enzyme for the anti-inflammatory compound PEA. The mean (SD) ∆Ct value for *PTGS2* relative to *NAPEPLD* for the control and OLP cases was 2.39 (1.08) and 1.29 (1.40) (*p* = 0.034, Welch’s *t*-test, *N* = 13), respectively (Figure 2). The difference between the mean values for the controls and the OLP patients, −1.10, corresponds to approximately a doubling in *PTGS2* expression relative to *NAPEPLD*. A similar change in the balance between *PTGS2* and *NAPEPLD* was seen in three human cell lines treated with TNF-α, a cytokine known to be involved in the pathogenesis of OLP (Figure 2).

### 3.2. NAE Levels in Control and OLP Biopsy Samples

**Figure 3:**
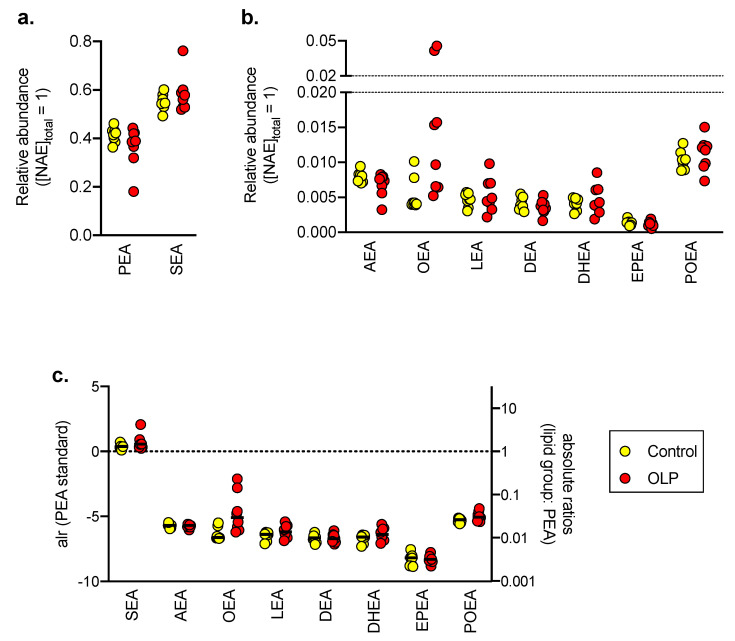
Composition of NAEs in control and OLP tissue biopsies. In panels (**a**,**b**), fractional contents, i.e., the concentration of lipid/combined concentration of all nine NAEs are shown for each case. Note the different scales on the y axis in the panels. In panel (**c**), the data are expressed as the additive log ratio (alr), with PEA as the denominator, i.e., ln(lipid/[PEA]), with the anti-logged absolute ratios shown on the right y axis. The solid horizontal bars represent the means. Abbreviations (when not already explained): LEA, linoleoyl ethanolamide; DEA, docosatetraenoyl ethanolamide; DHEA, docosahexaenoyl ethanolamide; EPEA, eicosapentaenoyl ethanolamide; POEA, palmitoleoylethanolamide.

A second cohort [25] comprised a series of eight controls and eight OLP patients where the biopsy samples had been embedded in Tissue-Tek and snap frozen (“PG/NAE cohort”). The analysis (ultra-performance liquid chromatography (UPLC) coupled to tandem mass spectrometry (MS/MS); see Methods) was run in both the positive (for the NAEs) and negative multiple reaction monitoring modes (for the arachidonic acid-derived oxylipins reported here and for the linoleic acid derivatives reported in [25]). Nine NAEs were detected in the control and OLP biopsy samples (Figure 3a,b). The embedding material did not contain detectable levels of the NAEs. However, the absolute amount of each sample in the embedded materials was not known, which means that absolute levels of the NAEs per unit weight are not possible to calculate. Instead, we have presented the data for each NAE as a proportion of the total NAEs recovered in each sample. Consistent with other tissues, PEA and SEA were prominent, together accounting for 92–97% of the total NAE recovered. OEA levels on the other hand only accounted for 0.53% of the total NAEs.

Statistical evaluation of the type of data shown in Figure 1a,b, termed compositional data, is not appropriate for the data per se, but log ratio transformations that render the data amenable to analysis have been developed. The simplest of these, the additive log ratio (alr) [30], has been used here using PEA as the reference (Figure 3c). A MANOVA using the alr data for SEA, AEA, OEA and POEA (using Pillai’s trace) gave V = 0.40 and F_4,1_ = 1.87, *p* = 0.19. This would indicate that the composition of these NAEs in the OLP biopsy samples is not significantly different from the control samples. Levels of the other four NAEs were deemed to be too low for statistical examination to be meaningful.

### 3.3. Oxylipin Levels in Control and OLP Biopsy Samples

**Figure 4:**
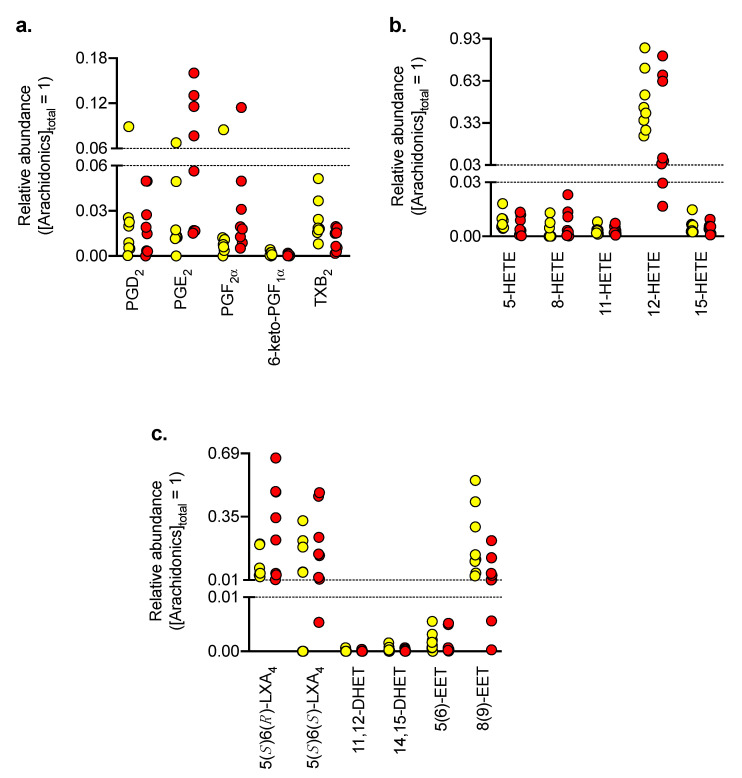
Composition of arachidonic acid-derived oxylipins in control (yellow) and OLP tissue (red) biopsies. The panels show fractional contents: (**a**) prostaglandins, (**b**) HETEs, and (**c**) other arachidonate derivatives, where the concentration of lipid/combined concentration of all sixteen oxylipins are shown for each individual case. Note the different scales on the y axis in the panel. Abbreviations (when not already explained): TXB_2_, thromboxane B_2_; DHET, dihydroxyeicosatetraenoic acid; EET, epoxyeicosatrienoic acid.

The relative abundances of PGs and other oxylipins in the biopsy samples (“PG/NAE cohort”) are shown in Figure 4. The most abundant oxylipins were 12-hydroxyeicosatetraenoic acid (12-HETE), 5(*S*)6(*R*)- and 5(*S*)6(*S*)-lipoxin A_4_ (LXA_4_). 

**Figure 5:**
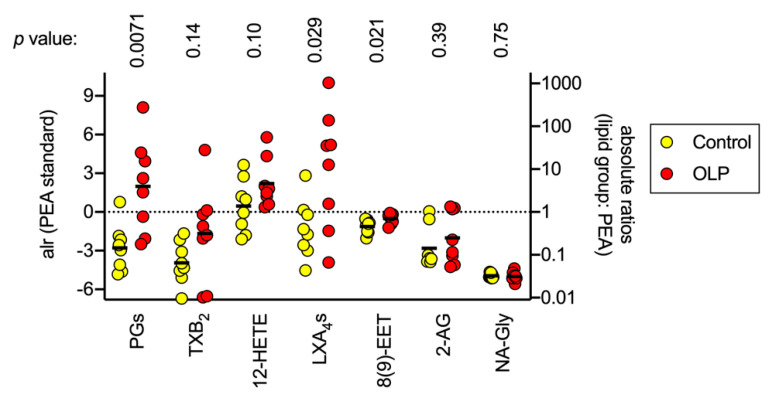
Additive log ratios (alr) with PEA as the denominator for PGs, TXB_2_, 12-HETE, LXA_4_s, 8(9)-EET, 2-AG and NA-Gly in the control and OLP biopsy samples. The additive log ratios (alr) with PEA as the denominator, i.e., ln(lipid/[PEA]) are given on the left y axis, with the corresponding anti-logged absolute ratios shown on the right y axis. The solid bars represent the means. The *p* values were determined by two-tailed *t*-tests not assuming equal variances. The critical value of *p* at a 5% false discovery rate is 0.0071.

For each of the three main PGs, one sample (different in each case) and three control samples for 5(*S*)6(*S*)-LXA_4_ had levels below the limit of detection, to which we assigned a priori zero values. This assignation rules out calculation of additive log ratios for the individual PGs and for LXA_4_s, but allows calculation for the sum of the three PGs and the two LXA_4_s. In Figure 5, the additive log ratios relative to PEA are shown for the combined PGs, TXB_4_, 12-HETE, LXA4s and 8(9)-EET, the other lipids having levels too low to be considered robust. Additionally, the runs in the positive multiple reaction monitoring mode detected the endocannabinoid 2-arachidonoylglycerol (2-AG) and the related lipid *N*-arachidonoylglycine (NA-Gly), which has been implicated the regulation of inflammatory pain [31]. A MANOVA for these lipids gave a significant effect of patient group (using Pillai’s trace, V = 0.78, F_6,9_ = 4.25, *p* = 0.012). For the individual lipids, the additive log ratios relative to PEA for the PGs were significantly greater for the OLP group than for the controls, and the *p* value (0.0071) was the same as the critical value of *p* assuming a 5% false discovery rate. The difference in the mean additive log ratios for the PGs for OLP and control was 4.77, corresponding to a fold difference of 27. Thus, in OLP, there is a large shift in the relative proportion of PGs to PEA towards the PGs. None of the other additive log ratios had *p* values below the critical value of *p* at a 5% false discovery rate.

## 4. Discussion

The aim of this study was to investigate the NAE synthetic and hydrolytic enzymes and NAE levels in comparison to PGs in OLP. At the outset, three weaknesses of this study should be stated: (1) sample sizes are small given the heterogeneous nature of OLP; (2) the qPCR data give gene expression rather than protein expression and/or enzyme activity measurements, which would be more informative; (3) the data on PGs and NAE are not absolute values but relative values. However, these weaknesses are offset by two main strengths: (1) the qPCR and PG/NAE data are taken from different, albeit small, cohorts and give mutually consistent data; (2) this study provides for the first time data on NAEs and their metabolic enzymes in OLP. Nonetheless, the small sample sizes mean that the present study should be considered as exploratory in nature, requiring future larger studies to confirm the findings, ideally not only in the entire heterogeneous population of OLP cases but also within the different OLP pathologies.

Given the anti-inflammatory properties of PEA [12], two scenarios in diseases can be considered: (1) a decreased level of PEA could be involved in the pathogenesis of the disease in question by resulting in a deficient level of an anti-inflammatory lipid. An example here would be in the mouse intestine, where croton oil-induced inflammation of the gut increases gastrointestinal transit and this is accompanied by a ~40% reduction in small intestine PEA levels. Administration of PEA reverses the effect of croton oil upon the gastrointestinal transit [32]. Alternatively, (2) the pathological state could produce a compensatory increase in PEA levels that might work to mitigate its consequences. An example here is the increase in PEA levels in the spinal cord in an animal model of multiple sclerosis for the animals showing spasticity, given the ability of exogenous PEA to reduce spasticity in this model [33]. 

With respect to the first alternative, such a deficit could result from a deficient synthesis and/or increased catabolism of PEA. At the level of mRNA, the present study did not find evidence of such changes. Indeed, in the case of *NAPEPLD*, if anything a significant increase was seen, with a *p* value less than 0.05, but higher than the critical value of *p* (0.0125) obtained using a 5% false discovery rate. The use (or not) of a correction for multiple testing for exploratory data such as the present study is less clear-cut than for confirmatory data [34], and for this reason we present unadjusted rather than adjusted *p* values to allow the readers to draw their own conclusions with respect to *NAPEPLD*.

The second suggested role of PEA is an increased level to mitigate the inflammation. Such a role may be occurring in post-partum uterine inflammation in cows, where increased *NAPEPLD* and decreased levels of *NAAA* and *FAAH* have been seen in endometrial samples [35]. There are a number of inflammatory markers that have been shown to be affected in OLP [1,2,3,36], but we have focused upon COX-2 simply because COX-2-derived prostaglandins and/or COX-2 itself are modulated by PEA treatment in vitro [15] and in vivo [14], and that *PTGS2*, COX-2 and PG levels are known to be increased in OLP [4,5,6,7]. Thus, Chankong et al. [7] reported that the immunohistochemical staining of epithelial COX-2 was approximately 1.4-fold higher in 25 OLP patients than the corresponding staining in 13 control samples. Unsurprisingly, staining was also seen in the inflammatory infiltrate in the OLP samples. Lysitsa et al. [4] reported that the expression of COX-2 relative to COX-1 was increased by approximately 2-fold in immunohistochemical staining of OLP epithelia (30 patients vs. 8 controls). In that study, the OLP patients were divided into three groups (patients with moderate or mildly active OLP (*N* = 9), patients with active or moderately active atrophic OLP (*N* = 12) and patients with mild or inactive atrophic OLP (*N* = 9)). In all three groups, COX-2 expression was increased compared to the controls, but there was no significant difference in expression between the different OLP groups [4]. The authors also found that the increase in COX-2 expression was similar for non-smokers and smokers [4]. 

If PEA acts to mitigate the inflammation, the increased expression of *PTGS2* and levels of PGs in OLP should be counterbalanced by the increased expression of *NAPEPLD* and levels of PEA. In other words, the ratios of *PTGS2*:*NAPEPLD* and PGs:PEA should not change much in OLP. In fact, the ratios were significantly higher in OLP than in the controls, suggesting that the increased expression of COX-2 is not matched by a similar mobilization of PEA. 

An important question concerns which of the inflammatory mediators found in OLP is responsible for the increased COX-2 expression. Relatively little work has been undertaken in this respect, but Singh et al., [37] reported a strong association of COX-2 immunoreactivity with both basement membrane integrity and the mast cell count in OLP. Mast cells are an important source of TNF-α, and so we were interested to determine whether the findings in OLP could be mimicked by treating cells with TNF-α. DU-145 prostate cancer cells respond to TNF-α treatment with an increased expression of *PTGS2*, of COX-2 and of PGE_2_ production [38] and this is accompanied by a decreased expression of *NAPEPLD* without change in *NAAA* or *FAAH* expression [29], thereby producing an imbalance in *PTGS2*/*NAPEPLD* (Figure 2). We investigated this further in the present study using two oral squamous carcinoma cell lines and found that TNF-α treatment also produced an imbalance in *PTGS2*/*NAPEPLD*. 

In addition to the main results discussed above, the novelty of the present study has resulted in a number of interesting basal observations. Thus, at the level of mRNA, the levels of *FAAH* in the control biopsies were ~5-fold lower than the corresponding levels of *NAAA.* This is similar to the situation in tongue biopsies [39]. The predominance of 12-HETE among arachidonic acid metabolites is consistent with the literature for human skin epidermal cells [40], and in healthy oral mucosa, Johnson et al. [41] reported levels of 12-HETE that were two orders of magnitude higher than those of PGE_2_, a finding consistent with the present data.

More unexpected was the finding that in the biopsy samples, OEA has a low relative abundancy among NAEs. In the control biopsy samples used in the present study, the mean relative content of OEA was only 0.53% of the total NAEs. In contrast, in human plasma [23], subcutaneous adipose tissue [42] and the trapezius muscle [43], OEA levels are generally of the same order of magnitude as of SEA and PEA. The simplest explanation for the low levels of OEA in the biopsy samples studied is that they reflect a low relative abundance of oleoyl species in the precursor lipids. To our knowledge, the abundance of these lipids this has not been investigated in oral mucosa.

In conclusion, we present for the first time data investigating the NAE system in relation to OLP. Our data suggest that PEA synthesis is not sufficiently mobilized in OLP to match the increased expression of COX-2. This lack of (or insufficient) mobilization of an endogenous PEA response to dampen the inflammation in OLP opens up the possibility that exogenous PEA may be a potentially useful treatment strategy for this disorder, given its anti-inflammatory properties (not least its ability to downmodulate mast cells [16] and to reduce levels of inflammatory cytokines secondary to its effects upon PPAR-α [12,13]; for review, see [44]) and given that current treatment strategies (primarily topical corticosteroid treatment) are suboptimal. PEA is currently available as a nutraceutical or food supplement in some European countries, is a constituent in a cream for dry skin, and is also used for skin conditions in veterinary medicine. In the clinic, PEA is very well tolerated and has shown to be effective in treating a range of inflammatory and pain conditions such as lumbosciatica [45], knee osteoarthritis [46], burning mouth syndrome [47] and multiple sclerosis [48]. These were oral treatments, but the effects of a PEA cream on 2456 patients with atopic eczema in a multinational, multicenter, observational, non-controlled, prospective cohort study have been studied [18]. The authors of that study reported that PEA was effective in treating mild to moderate eczema. It further reduced the need for glucocorticoid treatment. Clearly, a clinical trial of a suitable local PEA treatment would be of great interest in OLP.

## Figures and Tables

**Table 1:** Characteristics of the qPCR cohort.

Controls		OLP Patients			
Gender	Age	Gender	Age	OLP Type	Localization
M	69	M	67	reticular, atrophic, plaque, ulcerous	oral, genital
F	69	F	66	reticular, atrophic	oral, genital
M	49	F	63	reticular, atrophic, plaque	oral, genital
F	53	M	61	reticular, atrophic	oral, skin
F	50	M	58	reticular	oral
F	47	F ^a^	81	reticular, atrophic	oral
F ^a^	67	M	45	reticular, atrophic	oral, genital
F	45	F	63	reticular, atrophic	oral
F	44	F	73	reticular, atrophic	oral, genital
F	39	F	74	reticular, atrophic	oral, genital
M	66	F	64	reticular, atrophic	oral, genital
F	64	F	64	reticular, atrophic	oral, skin
F	61	M	67	reticular	oral
F	73	F ^b^	44	reticular	oral, genital
F	68	F	68	reticular, atrophic, ulcerous	oral

^a^ Samples not analyzed for technical reasons. ^b^ Patient used moist powder tobacco.

**Table 2:** Primer sequences for RT-qPCR.

Gene (Product).	Forward Primer (5′ to 3′)	Reverse Primer (5′ to 3′)	Efficiency
*RPL19* (Ribosomal protein 19)	CAC ATC CAC AAG CTG AAG GCA	CTT GCG TGC TTC CTT GGT CT	99%
*NAPEPLD* (NAPE-PLD)	ACT GGT TAT TGC CCT GCT TT	AAT CCT TAC AGC TTC TTC TGG G	99%
*PTGS2* (COX-2)	AGC AGG CAG ATG AAA TAC CAG	ACC AGA AGG GCA GGA TAC A	93%
*NAAA* (NAAA)	ATG GAG CGT GGT TCC GAG TT	AGG CTG AGG TTT GCT TGT CCT	99%
*FAAH* (FAAH)	CAC ACG CTG GTT CCC TTC TT	GGG TCC ACG AAA TCA CCT TTG A	99%

## Supplementary Materials

The following are available online at https://www.mdpi.com/2227-9059/8/11/481/s1, Figure S1: Effects of treatment of CAL27 human squamous cell carcinoma cells for 24 h with recombinant human TNF-α (10 ng/mL), IL-8 (10 (IL10) or 100 (IL100) ng/mL) and/or PEA (0, 3, 10 µM) upon mRNA levels of (**a**), *NAPEPLD*; (**b**), *PTGS2*; (**c**), *FAAH* and (**d**), *NAAA*., Figure S2: Effects of treatment of SCC-25 human squamous cell carcinoma cells for 24 h with recombinant human TNF-α (10 ng/mL), IL-8 (10 (IL10) or 100 (IL100) ng/mL) and/or PEA (0, 3, 10 µM) upon mRNA levels of (**a**), *NAPEPLD*; (**b**), *PTGS2*; (**c**), *FAAH* and (**d**), *NAAA*..
