# Supplementary material for: Relative Deficiency of Anti-Inflammatory N-Acylethanolamines Compared to Prostaglandins in Oral Lichen Planus
Source: Biomedicines. 2020 Nov 6;8(11):481. doi: 10.3390/biomedicines8110481 (PMC7694776; doi:10.3390/biomedicines8110481)
Supplement: Supplementary file 1 [file biomedicines-08-00481-s001.pdf]

Supplementary data

Relative deficiency of anti-inflammatory *N*-acyl-ethanolamines to prostaglandins in oral lichen planus

Linda Rankin [1], Sandra Gouveia-Figueira [2], Karin P. Danielsson[3] and Christopher J. Fowler [2,*]

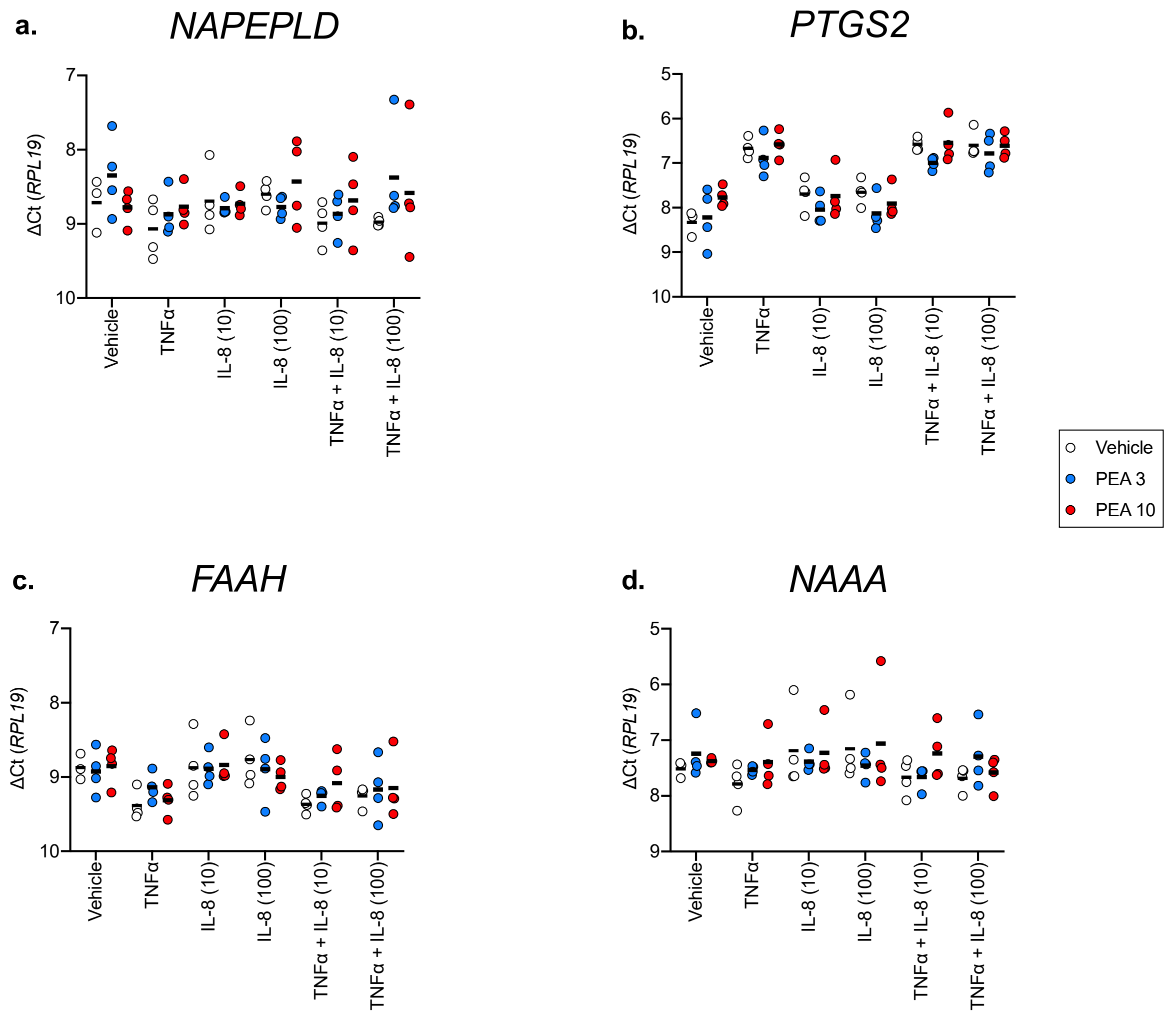

Figure S1. Effects of treatment of CAL27 human squamous cell carcinoma cells for 24 h with recombinant human TNF-α (10 ng/mL), IL-8 (10 (IL10) or 100 (IL100) ng/mL) and/or PEA (0, 3, 10 μM) upon mRNA levels of (**a**), *NAPEPLD*; (**b**), *PTGS2*; (**c**), *FAAH* and (**d**), *NAAA*. The figure shows scatterplots with the bars representing the means, N=3-4. The values in the absence of PEA were used to calculate the data shown in Figure 2 in the main article.

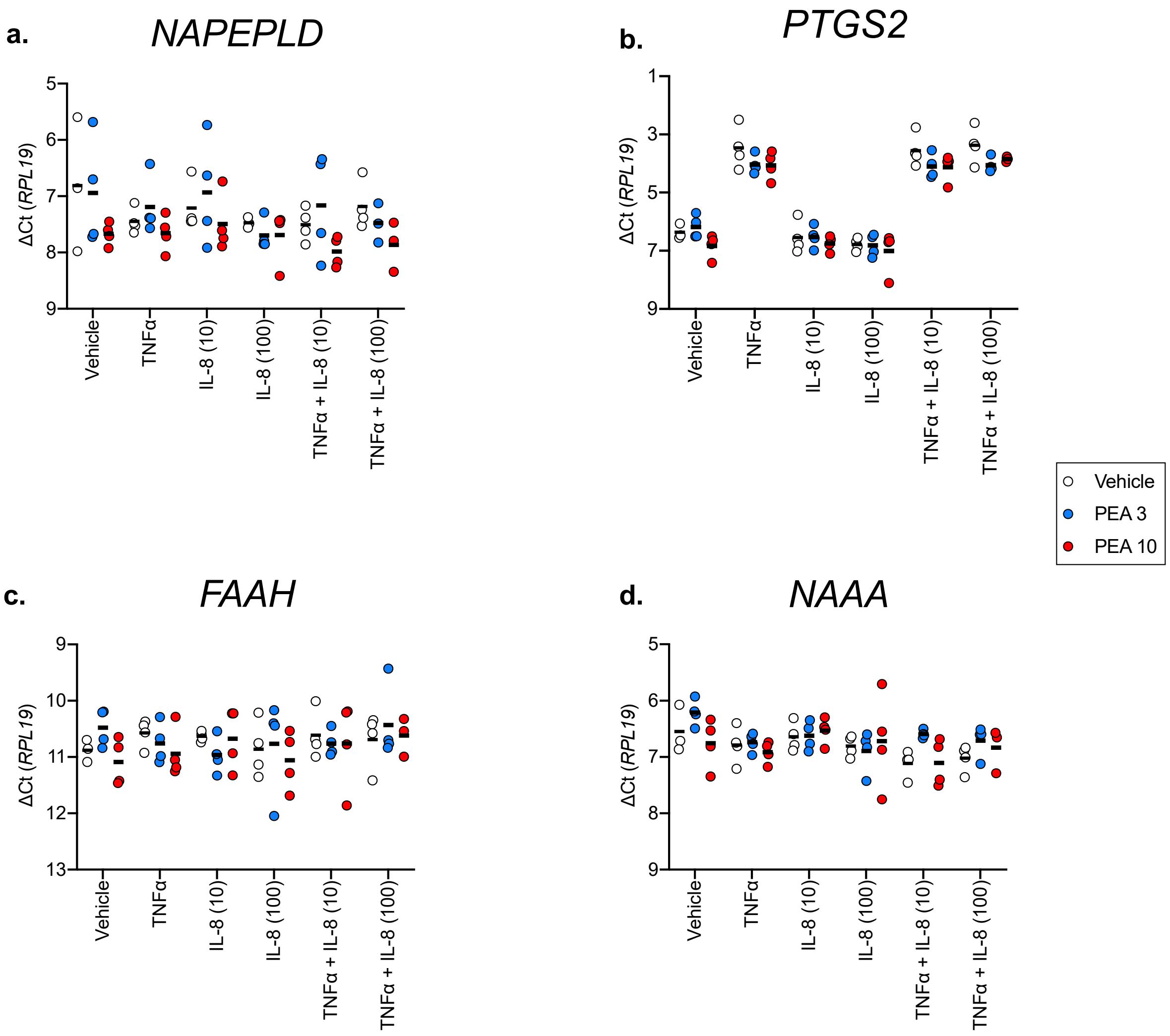

Figure S2. Effects of treatment of SCC-25 human squamous cell carcinoma cells for 24 h with recombinant human TNF-*α* (10 ng/mL), IL-8 (10 (IL10) or 100 (IL100) ng/mL) and/or PEA (0, 3, 10 μM) upon mRNA levels of (**a**), *NAPEPLD*; (**b**), *PTGS2*; (**c**), *FAAH* and (**d**), *NAAA*. The figure shows scatterplots with the bars representing the means, N=3-4. The values in the absence of PEA were used to calculate the data shown in Figure 2 in the main article.
